# Local Electrical Dyssynchrony during Atrial Fibrillation: Theoretical Considerations and Initial Catheter Ablation Results

**DOI:** 10.1371/journal.pone.0164236

**Published:** 2016-10-25

**Authors:** Pawel Kuklik, Benjamin Schäffer, Boris A. Hoffmann, Anand N. Ganesan, Doreen Schreiber, Julia M. Moser, Ruken Ö. Akbulak, Arian Sultan, Daniel Steven, Bart Maesen, Ulrich Schotten, Christian Meyer, Stephan Willems

**Affiliations:** 1 Department of Cardiology–Electrophysiology, University Hospital Hamburg, University Heart Center, Hamburg, Germany; 2 Flinders University School of Medicine and Department of Cardiovascular Medicine, Flinders Medical Centre, Adelaide, Australia; 3 Department of Cardio-thoracic Surgery, Maastricht University Medical Centre, Maastricht, The Netherlands; 4 Department of Physiology, Maastricht University, Maastricht, The Netherlands; 5 DZHK (German Centre for Cardiovascular Research), Partner Site Hamburg/Kiel/Lübeck, Hamburg, Germany; Universiteit Gent, BELGIUM

## Abstract

**Background:**

Electrogram-based identification of the regions maintaining persistent Atrial Fibrillation (AF) is a subject of ongoing debate. Here, we explore the concept of local electrical dyssynchrony to identify AF drivers.

**Methods and Results:**

Local electrical dyssynchrony was calculated using mean phase coherence. High-density epicardial mapping along with mathematical model were used to explore the link between local dyssynchrony and properties of wave conduction. High-density mapping showed a positive correlation between the dyssynchrony and number of fibrillatory waves (R^2^ = 0.68, p<0.001). In the mathematical model, virtual ablation at high dyssynchrony regions resulted in conduction regularization. The clinical study consisted of eighteen patients undergoing catheter ablation of persistent AF. High-density maps of left atrial (LA) were constructed using a circular mapping catheter. After pulmonary vein isolation, regions with the top 10% of the highest dyssynchrony in LA were targeted during ablation and followed with ablation of complex atrial electrograms. Catheter ablation resulted in termination during ablation at high dyssynchrony regions in 7 (41%) patients. In another 4 (24%) patients, transient organization was observed. In 6 (35%) there was no clear effect. Long-term follow-up showed 65% AF freedom at 1 year and 22% at 2 years.

**Conclusions:**

Local electrical dyssynchrony provides a reasonable estimator of regional AF complexity defined as the number of fibrillatory waves. Additionally, it points to regions of dynamical instability related with action potential alternans. However, despite those characteristics, its utility in guiding catheter ablation of AF is limited suggesting other factors are responsible for AF persistence.

## Introduction

Although pulmonary vein isolation (PVI) [1] is the cornerstone of catheter ablation of paroxysmal AF, the optimal approach to catheter ablation of persistent atrial fibrillation (persAF) is yet to be resolved [2]. Current guidelines recommend more extensive linear or complex fractionated electrogram ablation in persAF. However, a series of recent randomized controlled trials have failed to demonstrate an additional clinical benefit of the application of these empirically derived approaches beyond PVI [3–5]. In this context, there is a significant interest in the development of novel quantitative electrogram approaches to guide persAF ablation [6, 7]. To date, reports on new electrogram-based strategies in AF ablation have frequently provided relatively limited detail regarding technical details of implementation [8], leading to challenges in reproducibility and validation [9].

In this study, we sought to conduct a comprehensive computer to catheter laboratory study evaluating the derivation and evaluation of local electrical dyssynchrony as a novel parameter to guide ablation in persistent AF. The study consists of three parts. Part 1 of the study explores the derivation and mathematical modeling of dyssynchrony in AF. Part 2 investigates the application of dyssynchrony in epicardial recordings of AF in humans. Part 3 involves a pilot investigation of dyssynchrony-guided adjunctive ablation in humans.

## Methods

### Definition and derivation of local electrical dyssynchrony

**Fig 1:**
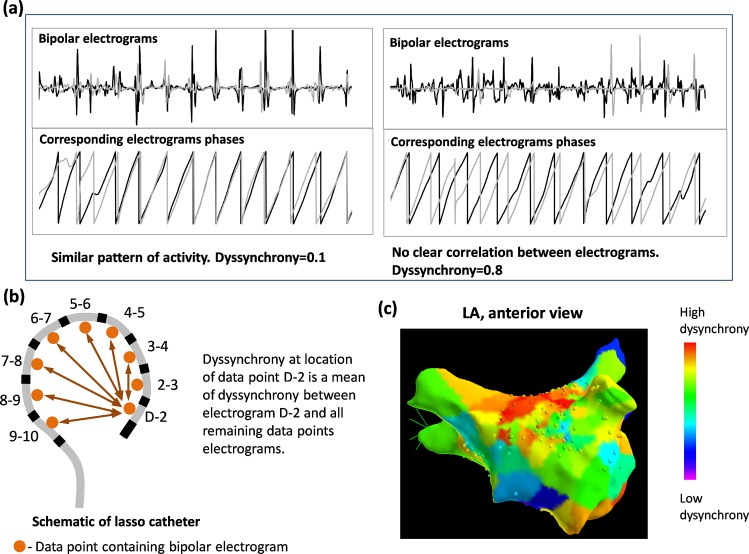
Definition of local electrical dyssynchrony. **(a)** An example of pairs of electrograms (first electrogram in black, second electrogram in gray) with low (left) and high (right) dyssynchrony level. At the top, original electrograms and below corresponding electrograms phases used to compute the dyssynchrony parameter. (b) Schematic of the circular catheter and calculation of dyssynchrony of a given data point ('D-2' in this example). (c) 3D map with color coded value of electrical dyssynchrony.

Electrical dyssynchrony between two electrograms was quantified using concept of mean phase coherence [10], defining dyssynchrony as:

$$Dyssynchrony=1-1/T|\sum_{t=1}^{t=T}\exp(i(\varphi_{1}(t)-\varphi_{2}(t))|$$ (1)

where t denotes time, T is the length of the electrogram segment taken into the analysis (8 s in our study), ϕ_1_(t) and ϕ_2_(t) are electrograms phases at time point t. Electrogram phase was reconstructed using Hilbert transform preceded by sinusoidal recomposition as described in [11]. Dyssynchrony value varies between 0 denoting full 1:1 synchronization (constant phase shift between electrical activity in both electrograms) and 1 denoting a total lack of coherence of electrical activity. Examples of low and high dyssynchrony between two electrograms are shown in Fig 1A. An example of the region of high dyssynchrony is shown in Fig 1C. See S1 Text for details on phase compuation.

### High-density epicardial mapping

**Fig 2:**
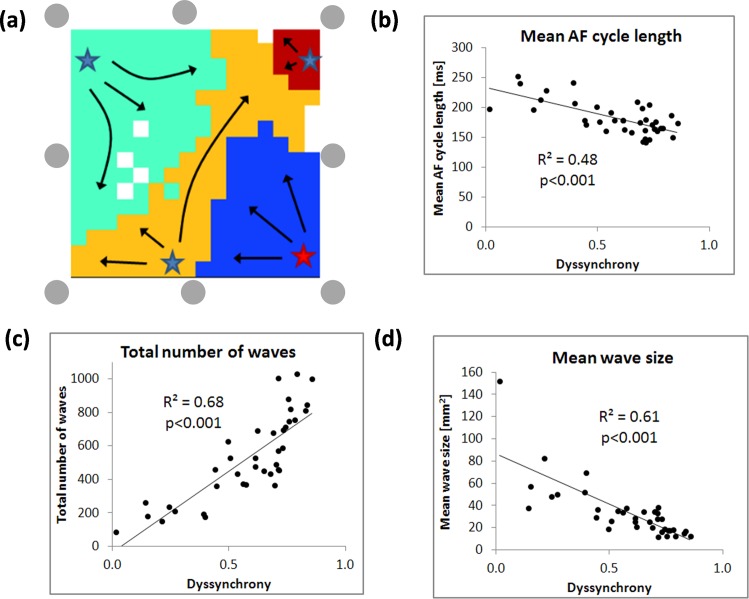
Correlation of electrical dyssynchrony and wave characteristics derived from high-density epicardial mapping. (a) An example of individual waves identification (each wave denoted in a different color) in mapped field. Electrical dyssynchrony was calculated using a subset of electrodes with positions indicated by gray circles resulting in comparable layout as in a case of the circular catheter. Relation of the mean dyssynchrony between given subset of electrodes and mean AF cycle length (b), the total number of waves detected during whole recording (c) and the mean size of detected waves (d). Each point in the plot represents a mean value from single 10 s recording. Each point in the graphs (b-c) denotes an estimate from a single recording.

Direct contact mapping was performed in 21 AF patients undergoing open chest surgery in Maastricht University Medical Centre. 11 pts were in paroxysmal AF and 9 pts were in persistent AF. Rectangular plaques (16x16 electrodes, 1.5 mm inter-electrode spacing) were placed on the epicardial surface of the LA posterior wall between the pulmonary veins and the RA free wall. Atria were mapped sequentially for 10 s at each site. Electrograms were sampled at 1 kHz. Individual waves were identified by an algorithm previously described [12]. For each map, electrical dyssynchrony was calculated using a subset of electrodes located at the corners and the middle points of the edges (gray circles in Fig 2A) to mimic the layout of the circular catheter. Dyssynchrony per recording was calculated as a mean dyssynchrony between all pairs of electrodes. Each recording was characterized by mean AF cycle length, wave size and a total number of waves.

### Panfilov-Tusscher mathematical model of dynamically unstable wave conduction

One of the mechanisms of wave conduction driving AF is the presence of dynamically unstable area (e.g. high degree of electrical alternans) causing continuous wave break and fibrillatory conduction[13, 14]. Dynamically unstable areas would continuously disrupt wave conduction in the surrounding, generating short-lived pivoting waves. To model this scenario, we used a Panfilov-Tusscher model [15] which, due to its simple formulation, allows straightforward control of the dynamical stability of the wave conduction. We used a rectangular grid of 100x100 diffusively coupled simulation nodes (diffusion coefficient set to 0.2 mm^2^/ms). Space step was set to 0.6 mm and time step to 0.02 ms. The system was integrated using forward Euler scheme with no-flux boundary conditions. Since we did not aim to model detailed electrophysiology of the atria but rather show a specific mechanism, we used arbitrary units. Based on the study by ten Tusscher and Panfilov [15], we used following base parameters of the model: a = 0.1, k = 8, ε = 0.01, b = 0.1, μ_1_ = 0.2, μ_2_ = 0.2.

In order to model an inhomogeneous substrate for AF we varied parameter μ_1_ in the model which strongly influences steepness of the restitution curve. We introduced a random distribution of μ_1_ (following the approach used in [16]) which led to the presence of localized areas of high dynamical instability (resulting in spiral wave breakup) surrounded by dynamically stable areas. The μ1 landscape was modified as follows: for each grid element {i, j}, the μ_1_ values within a circular region of radius R were multiplied by a coefficient c with the probability p where c was defined as:

$$c(r)=I\frac{r}{R}+1-I$$ (2)

where I determined the amplitude of the decrease and R is the range of the decrease. In this study, we used I = 0.04, R = 10 mm, p = 0.02 to obtain a spatial scale of inhomogeneities comparable with wave size. See S1 Movie to see how dynamical instability leads to a wave break in such prepared system.

We used this simplified model only to illustrate the link between dyssynchrony and regions maintaining AF due to electrical instability. There are several mechanisms thought to play an important role in AF maintenance (e.g. fibrosis, automaticity, heterogeneity of effective refractory period). However, exploration of all potential factors is beyond the scope of this work and we decided to focus on selected one (heterogeneity of electrical alternans) to demonstrate how dyssynchrony can point to regions critical to AF maintenance.

### Catheter ablation: study population and electrophysiological study

The AF ablation group included 18 patients (mean age 59±9 years; 12 male) undergoing catheter ablation for persistent AF between June and December 2013 at University Heart Center in Hamburg (see S2 Text for inclusion criteria along with patient characteristics). Antiarrhythmic drugs, with the exception of amiodarone, were ceased at least 5 half-lives before the procedure. The study was approved by the institutional ethical committee, and all patients gave written informed consent.

Local electrograms were recorded using the circular mapping catheter. Up to nine individual data points were collected per single recording site (depending on catheter contact with the atrial wall). Each data point corresponds with a recording of bipolar electrogram of 8 s duration. Data points with bipolar voltage amplitude below 0.1 mV were removed from analysis to avoid non-contact electrograms. The catheter was positioned at multiple sites aiming for complete coverage of the LA. Electrograms were sampled at 2034 Hz. No interpolation was used in the creation of 3D color-coded maps of dyssynchrony. Value at each point of the surface was assigned according to the value of the closest data point. Details of the electrophysiological study are described in S2 Text.

### Assessment of the dyssynchrony using circular mapping catheter

Dyssynchrony at a given data point was defined as mean dyssynchrony between this data point an data points recorded at remaining electrodes of mapping catheter (Fig 1B). Such formulation allowed us to identify points in LA at which electrical activity was out of synchrony with surrounding area. Electrogram fractionation was quantified using Complex Fractionated Atrial Electrogram (CFAE) definition based on the algorithm as defined in NavX mapping system with following settings: refraction time 50 ms, peak-to-peak sensitivity 0.05 mV, duration 10 ms and minimum slope threshold 0.02 mV/ms [17].

### Radiofrequency ablation and follow-up

Regions with the top 10% of high dyssynchrony were marked on 3D LA geometry in the NavX system as a guide to ablation (see Fig 1C). In case AF did not terminate after ablation of dyssynchrony regions, stepwise ablation of LA, right atria (RA) and coronary sinus (CS) was performed as previously described [18]. Details of radiofrequency ablation are described in S2 Text. The procedural end point was AF termination to sinus rhythm. In case of termination to atrial tachycardia, ablation was continued to achieve sinus rhythm. When AF termination was not achieved, electrical cardioversion was performed after a maximum of 6 h or 5 L of fluid intake. Patients were seen in our outpatient clinic for follow-up at 3, 6, 12 and 24 months. 24h Holter-ECG-recordings were assessed. An arrhythmia recurrence was defined as any episode of atrial tachycardia (AT) or AF **>** 30 s, documented on ECG recordings, rhythm strips or Holter-ECG.

### Statistical analysis

Results are expressed as mean±SD. Statistical analysis was performed using 1-way ANOVA with Bonferroni correction if applicable. *p<*0.05 was considered statistically significant.

The study was approved by the ethical committee METC of Academic Hospital Maastricht/Maastricht University and Ethics Committee of the University Heart Center Hamburg.

## Results

### Correlation of dyssynchrony with wave conduction parameters: high-density epicardial mapping

High-density epicardial mapping of AF revealed the presence of multiple wavelets propagating within the mapped field (as previously described in [12]). An example of the identification of individual waves is shown in Fig 2A. Analysis of the dyssynchrony between electrograms at the corners and edges of the plaque (see gray circles in Fig 2A) demonstrated that higher dyssynchrony was related to a lower AFCL, a greater number of waves and a smaller wave size. There was a significant correlation between mean dyssynchrony and mean AF cycle length (R^2^ = 0.48, p<0.001; see Fig 2B), the total number of waves (R^2^ = 0.68, p<0.001; see Fig 2C) and the mean size of detected waves (R^2^ = 0.61, p<0.001; see Fig 2D).

### Dyssynchrony guided ablation of dynamically unstable regions: mathematical model

**Fig 3:**
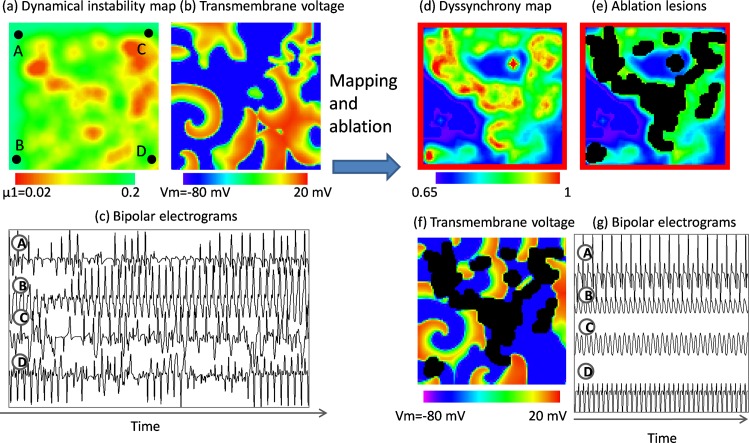
Electrical dyssynchrony in the mathematical model. (a) A mathematical model of AF based on the presence of regions of dynamical instability leading to wave break (red regions). (b) Map of transmembrane voltage showing individual waves in red. (c) Bipolar electrograms measured at corners of the plaque demonstrating irregularity of morphology and temporal sequence of activations. (d) Dyssynchrony map reconstructed after simulation for 40 cycles. (e) Regions of high dyssynchrony (in red) became a target of virtual ablation (black regions). (f) The result of virtual ablation on the propagation pattern. (g) Ablation resulted in regularization as evidenced by electrical activity measured at corners of the plaque.

Virtual ablation (ablation in the mathematical model of AF) of high dyssynchrony regions led to regularization of wave conduction. For simplicity, we just present a representative case (see Fig 3). Distribution of the parameter controlling dynamical stability in the model is shown in Fig 3A (with dynamically unstable regions leading to wave break in red). In such system, initiated spiral wave degenerated into irregular activity (Fig 3B) as demonstrated by bipolar electrograms recorded at corners of the plaque (Fig 3C). After simulation for 40 cycles, a dyssynchrony map was calculated showing general resemblance to the layout of parameter controlling dynamical stability (Fig 3D). Areas of high dyssynchrony (in red) became a target for virtual ablation (removing specified regions from the simulation mesh). Ablation led to the organization of the conduction—two stable spiral waves remained with a passive 1:1 conduction to the rest of the plaque (Fig 3F). Organization was demonstrated by stable morphology and temporal sequence of bipolar electrograms recorded at the corners (Fig 3H). As a numerical control experiment, we performed ablation in the areas of low dyssynchrony. Despite roughly the same area of ablation lesions, irregular wave conduction continued (see S2 Fig).

### Dyssynchrony mapping and catheter ablation of persistent AF

**Fig 4:**
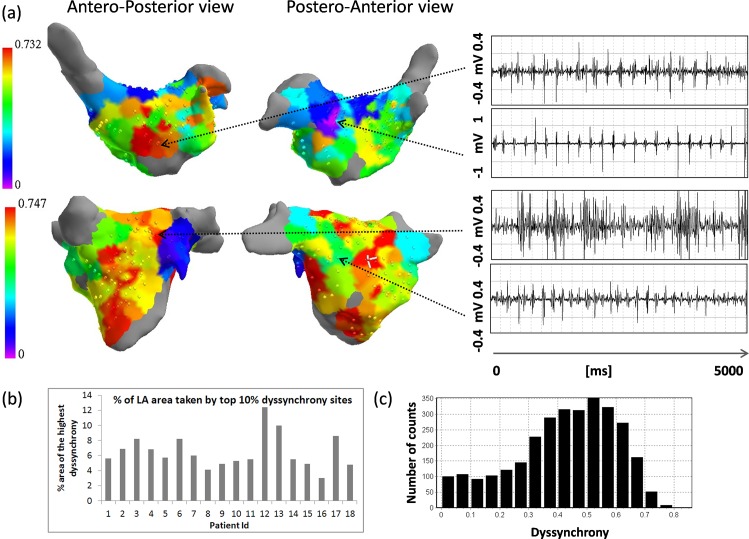
Example dyssynchrony maps with electrograms from areas of high and low dyssynchrony. (a) Dyssynchrony maps. Dyssynchrony level is color-coded with purple corresponding with the lack of dyssynchrony (regular local pattern of activity) and red corresponding to 90% of the maximum value of dyssynchrony in given map. Red areas became the targets of ablation. (b) Percentage of the assigned area taken by top 10% of dyssynchrony per patient. Distribution of all dyssynchrony values at all collected all data points (c) (bin size of 0.05).

AF induction was performed in 7 pts (39%). On average, 313±47 data points were collected during LA mapping. After removing low voltage points (<0.1 mV), 211±39 data points remained. Out of these, 175±46 data points were included in the analysis (points from single recording were included only if at least 4 points were in good contact to ensure a sufficient number of neighbors is present for dyssynchrony calculation). An example dyssynchrony maps are shown in Fig 4A. Top 10% dyssynchrony area covered on average 6.5±2.3% of the mapped LA surface (see Fig 4B). All dyssynchrony maps are shown in S1 Fig. Pulmonary vein isolation was performed successfully in all patients. Ablation at high dyssynchrony sites resulted in acute termination in 7 pts (39%), slowing down by >20 ms or occurrence of periods of regularization (transient temporal alignment of activations in catheters in left atrial appendage, coronary sinus and right atria) in 4 pts (22%) and had no effect in remaining 7 pts (39%). Following stepwise defragmentation resulted in termination in additional 4 pts reaching overall acute termination rate of 61%. At one year follow-up, 65% pts were AF free. Procedural characteristics and results are presented in S2 Text.

### Correlation of dyssynchrony with electrogram amplitude and fractionation

**Fig 5:**
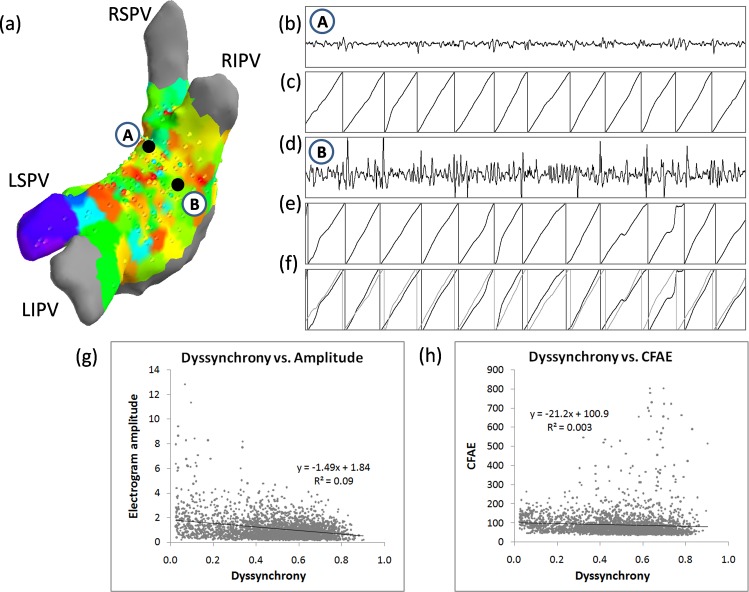
Correlation of dyssynchrony and electrogram amplitude and fractionation. An example of low voltage electrogram and fractionated electrogram having a low degree of dyssynchrony (0.46 for full 8 s segment). (a) Locations of the points on the LA geometry. (b) and (d) 2 s fragments of electrograms. (c) and (e) Phases corresponding to both electrograms. (f) Superposition of the phases of both electrograms showing a high degree of overlap denoting low dyssynchrony. (g) The relationship between dyssynchrony and electrogram amplitude for all data points. (g) The relationship between dyssynchrony and electrogram fractionation for all data points (N = 2986).

In order to explore the link between dyssynchrony and electrogram morphology, we correlated dyssynchrony with electrogram amplitude and the degree of fractionation. Electrogram amplitude was calculated as a mean of peak to peak value within 1 s windows (8 windows in total in single electrogram). Results are shown in Fig 5G–5H. The correlation was significant but very weak: R^2^ = 0.09 (p<0.001) with electrogram amplitude and R^2^ = 0.003 (p<0.005) with CFAE parameter).

### Temporal stability of dyssynchrony

**Fig 6:**
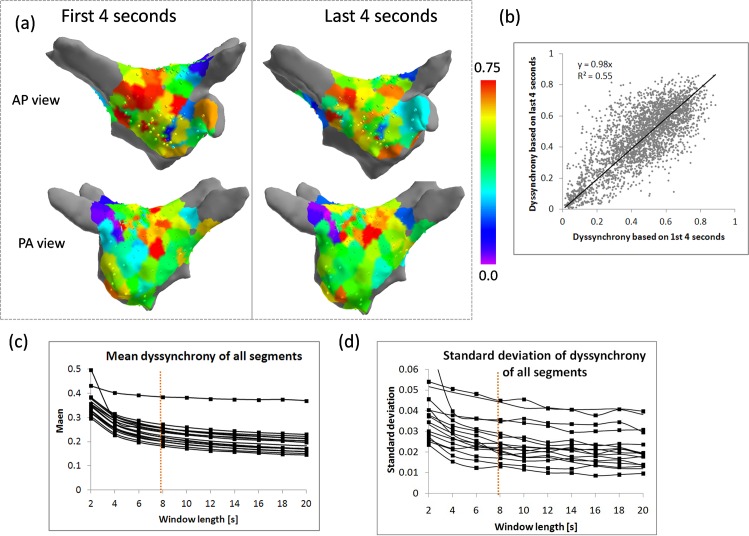
Temporal stability of dyssynchrony measure. (a) Example maps of local dyssynchrony in first and last 4 s of recorded segment. (b) Correlation between local dyssynchrony in first and last 4 s of recorded segment (all data points). (c) The dependency of mean (c) and standard deviation (d) of dyssynchrony for different lengths of the electrogram used in the analysis.

Example map of dyssynchrony calculated on first and last 4 s of the recording is shown in S3 Fig. Locations of the high dyssynchrony areas showed a good agreement in general, but there were instances when a high dyssynchrony region which was present in 8 s map was not present in maps calculated using 4 s segments (e.g. a region of high dyssynchrony next to the appendage in first row in S3 Fig). The overall correlation between dyssynchrony based on first and last 4 s resulted in R^2^ = 0.55, p<0.001 (see Fig 6B).

In order to explore whether 8 s window length is justified, we used 60 s recordings from the coronary sinus. We calculated dyssynchrony between all 9 bipolar electrograms using a sliding window of length varying between 2 and 20 s. At short window lengths (shorter than 6 s), mean and standard deviation were markedly higher. At 8 s, both mean and standard deviation reach a more steady level with a slow drift towards smaller values (Fig 6C and 6D).

## Discussion

We sought to provide a comprehensive derivation and evaluation of electrical dyssynchrony as a novel parameter to guide ablation in persistent AF. The principal findings of the study are:

1. In the mathematical model of AF, local dyssynchrony is colocalized with regions of steep APD restitution, with virtual ablation of regions of high dyssynchrony resulting in the termination of simulated AF.
2. In epicardial mapped AF in humans, regions of high dyssynchrony are correlated with the number of individual activation waves
3. In a pilot study of adjunctive dyssynchrony-guided ablation in human persistent AF; no additional benefit were demonstrated.

### Derivation of Dyssynchrony and Mathematical Model Findings

The first part of the study sought to explicitly present the derivation and validation of dyssynchrony mapping in a virtual mathematical model of AF. Virtual ablation in a mathematical model of AF reproduced two characteristic phenomena observed in the clinical setting: (i) occurrence of cardioversion (or lack of it) after ablation of the significantly large area of the atria and (ii) regularization of the electrograms. In our model successful virtual ablation was related with ablation of the areas with the steepest APD restitution, as revealed by dyssynchrony map (see Fig 3D). Presented model is very simplified (both in terms of action potential kinetics and geometry). However, we feel it provides an interesting insight into the link between dyssynchrony, APD restitution, and ablation-related AF termination.

### Epicardial Mapping of Dyssynchrony

In part 2 of the study, the correlation between dyssynchrony and fibrillatory wave characteristics was investigated. We found a positive correlation between dyssynchrony and fibrillation wave characteristics obtained during high-density epicardial mapping (number of waves and mean wave size; see Fig 2C and 2D). The number of waves is considered as an excellent surrogate parameter of AF complexity [19]. Since the area mapped using the epicardial electrode plaque (2.25x2.25 cm square) is of comparable size with the area captured by circular catheter, this result suggests that dyssynchrony may be a good estimator of the number of waves in encompassed area. Thus, local electrical dyssynchrony seems to be a robust tool for quantification of local AF complexity.

### Correlation of dyssynchrony with electrogram amplitude and fractionation

We found a very weak correlation between dyssynchrony and CFAE and electrogram amplitude (see Fig 5G and 5H). There were instances when high dyssynchrony was related with both high and low voltage (see Fig 5A–5F). Even low amplitude electrograms which would result in low fractionation (since the voltage would often be below detection threshold) can exhibit a low or high degree of dyssynchrony with surrounding areas (see Fig 5A–5F). This suggests that dyssynchrony measure is not merely a correlate of low amplitude or electrogram fractionation, but rather it provided additional information on electrogram properties.

### Temporal stability of dyssynchrony

AF mapping based on various parameters derived from AF electrograms is often questioned concerning the temporal stability of obtained maps. We generally found good agreement between dyssynchrony maps in the first and last 4 s of the recorded segment. However, in some cases, we found marked differences, especially in regions with low sampling density (for example see first row in S3 Fig).

We found an 8 s window sufficient to obtain an estimate of the dyssynchrony level. Segments lengths longer than 8 s did not result in significantly different estimates (see Fig 6C and 6D).

### Catheter Ablation of Regions of Dyssynchrony

In part 3 of the study, a preliminary investigation of the impact of adjunctive ablation of regions of dyssynchrony. Ablation of high dyssynchrony areas resulted in a modest incremental slowing of AFCL than PVI (17±22 ms increase vs. 7±22 ms). Importantly, however, the rate of acute AF termination (41%) seen in this pilot study is not significantly improved over recent persAF clinical trials. Similarly, the long-term freedom from arrhythmia in this pilot showed no advantage to with adjunctive dyssynchrony ablation.

### Interpretation and Clinical Implications

The optimal approach to the ablation of persAF is yet to be defined and remains a clinically immensely significant problem. A variety of empirically-derived methods, such as linear and fractionated electrogram ablation, has been utilized in the ablation of persAF, which have been based on clinical ablation experience in individual laboratories. To date, none has gained universal acceptance, in part because these methods have been difficult to uniformly validate and reproduce in follow-up clinical investigations [3, 5, 20]. Thus far, the conventional paradigm in the ablation of persAF has been for follow-up computational and mapping studies to be conducted to investigate and understand the mechanistic basis for empirically-derived ablation strategies [21].

In our study, we sought to invert the challenge the conventional paradigm in the development of a new dyssynchrony-based approach to persAF ablation. Despite, initially promising computational and mapping results supporting the concept of and mechanism of dyssynchrony based ablation, application of the technique in humans failed to demonstrate a definite clinical advantage over traditional PVI. Although this aspect of the investigation could be interpreted as a negative finding, we believe that detailed presentation and publication of the methods and approaches utilized are necessary to advance scientific understanding of persAF and ablation outcomes. The methodology employed have been presented explicitly and in detail to enable the technique to be reproduced and studied in future investigations.

AF complexity can be approached from many angles, giving partial insights into its mechanism. (e.g. spectral analysis to map the rate of activity at different regions of the atria or phase singularity mapping to identify pivoting waves). It is likely, that combination of various mapping approaches may be required to characterize its dynamics fully. This manuscript specifically contributes to this problem a link between dyssynchrony and AF complexity and the effect of ablation at high dyssynchrony sites.

## Conclusions

Local dyssynchrony of local electrical activity during AF is linked with the number of individual waves. However, ablation at areas of high dyssynchrony was not associated with improved persAF termination or long-term freedom of AF in humans. Detailed bench to bedside investigations will be essential to the development of future persAF ablation strategies.

## Study Limitations

The limitations of the study are as follows: (i) Stepwise ablation including both atria and the coronary sinus followed the ablation of dyssynchrony regions. Therefore, only acute termination result is specific to dyssynchrony ablation, and follow-up is a combined effect of dyssynchrony and stepwise ablation. (ii) As this is a pilot study, we included a small number of patients. (iii) We used a very simple mathematical model of AF with respect to geometry and action potential kinetics. In our study, we wanted to link dyssynchrony with specific patterns/types of conduction, which are easier to model and control in simple models than in complex, realistic models. (iv) Due to the limitation of the export length in NavX system, we were able to analyze electrograms of maximum 8 s length.

## Supporting Information

S1 Fig Dyssynchrony maps of all patients. (PDF)

S2 Fig The effect of ablation of non-dyssynchronous regions. The effect of virtual ablation of low dyssynchrony regions (marked in black in panel (a)). Map of transmembrane voltage showing individual waves in red (b). Conduction pattern continued to be irregular as evidenced by changing morphology of the bipolar electrograms recorded at the corners of the plaque (c).
(PDF)

S3 Fig Examples of temporal stability of dyssynchrony map (5 patients). Dyssynchrony map (anterior and posterior views) of whole 8 s electrograms segment on the left side. Map of the first 4 s segment in the middle and the map of last 4 s on the right side of the panel.
(PDF)

S1 File Figures data. (ZIP)

S1 Movie Spiral wave breakup in mathematical model. (AVI)

S1 Text Details on computation of electrogram phase. (PDF)

S2 Text Catheter ablation study details. (PDF)

## Abbreviations

AF: Atrial Fibrillation
LA: Left Atrium
PVI: Pulmonary Veins Isolation
CFAE: Complex Fractionated Atrial Electrogram
