# Supplementary material for: Local Electrical Dyssynchrony during Atrial Fibrillation: Theoretical Considerations and Initial Catheter Ablation Results
Source: PLoS One. 2016 Oct 25;11(10):e0164236. doi: 10.1371/journal.pone.0164236 (PMC5079563; doi:10.1371/journal.pone.0164236)
Supplement: S1 Fig — (PDF) [file pone.0164236.s001.pdf]

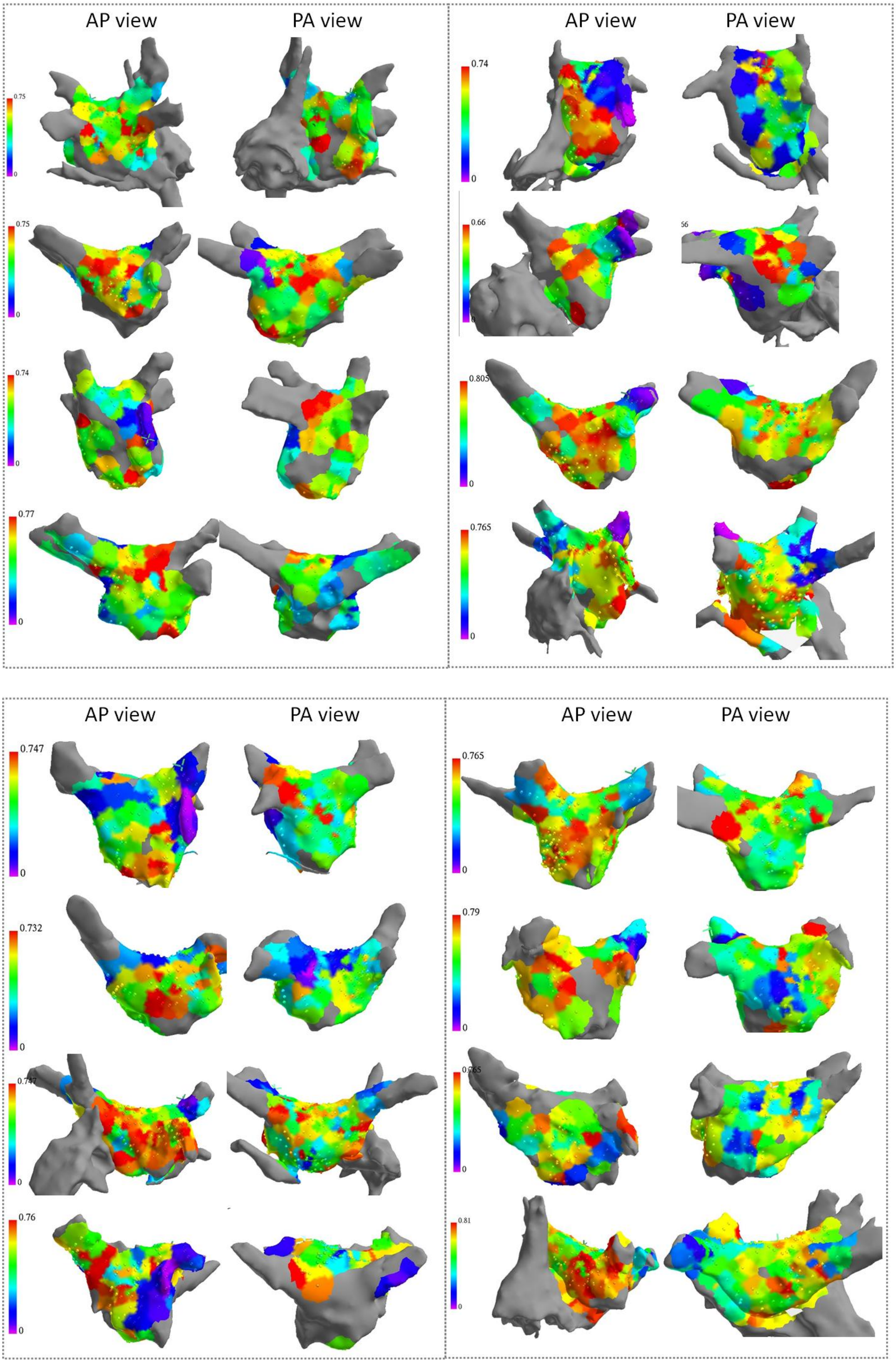

S1 Fig. Dyssynchrony maps of all patients (except the map of pt no. 17 which is shown as a bottom map in Fig 5 in the main manuscript).
