# Supplementary material for: Local Electrical Dyssynchrony during Atrial Fibrillation: Theoretical Considerations and Initial Catheter Ablation Results
Source: PLoS One. 2016 Oct 25;11(10):e0164236. doi: 10.1371/journal.pone.0164236 (PMC5079563; doi:10.1371/journal.pone.0164236)
Supplement: S2 Fig — The effect of virtual ablation of low dyssynchrony regions (marked in black in panel (a)). Map of transmembrane voltage showing individual waves in red (b). Conduction pattern continued to be irregular as evidenced by changing morphology of the bipolar electrograms recorded at the corners of the plaque (c). (PDF) [file pone.0164236.s002.pdf]

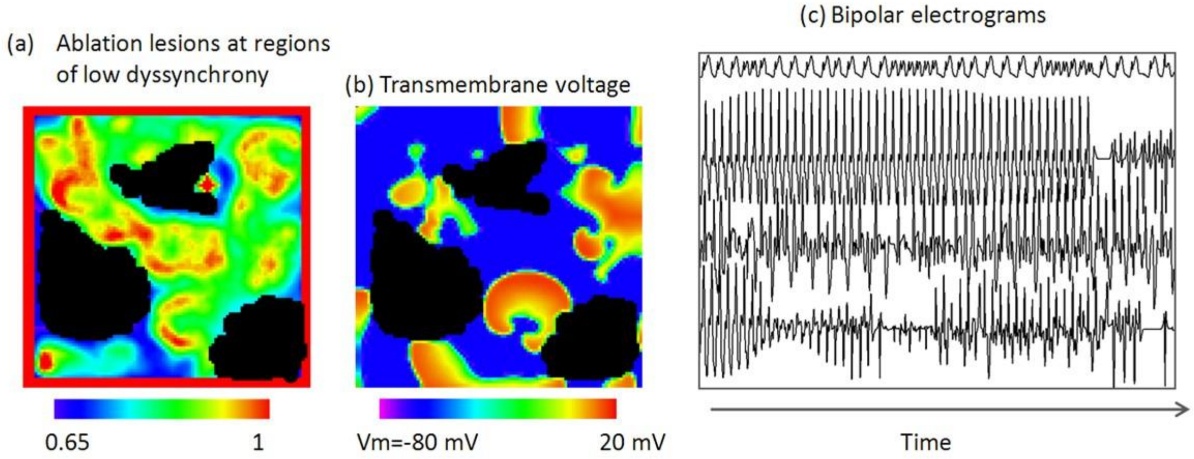

S2 Fig. The effect of virtual ablation of low dyssynchrony regions (marked in black in panel (a)). Map of transmembrane voltage showing individual waves in red (b). Conduction pattern continued to be irregular as evidenced by changing morphology of the bipolar electrograms recorded at the corners of the plaque (c).
