# Supplementary material for: Local Electrical Dyssynchrony during Atrial Fibrillation: Theoretical Considerations and Initial Catheter Ablation Results
Source: PLoS One. 2016 Oct 25;11(10):e0164236. doi: 10.1371/journal.pone.0164236 (PMC5079563; doi:10.1371/journal.pone.0164236)
Supplement: S3 Fig — Dyssynchrony map (anterior and posterior views) of whole 8 s electrograms segment on the left side. Map of the first 4 s segment in the middle and the map of last 4 s on the right side of the panel. (PDF) [file pone.0164236.s003.pdf]

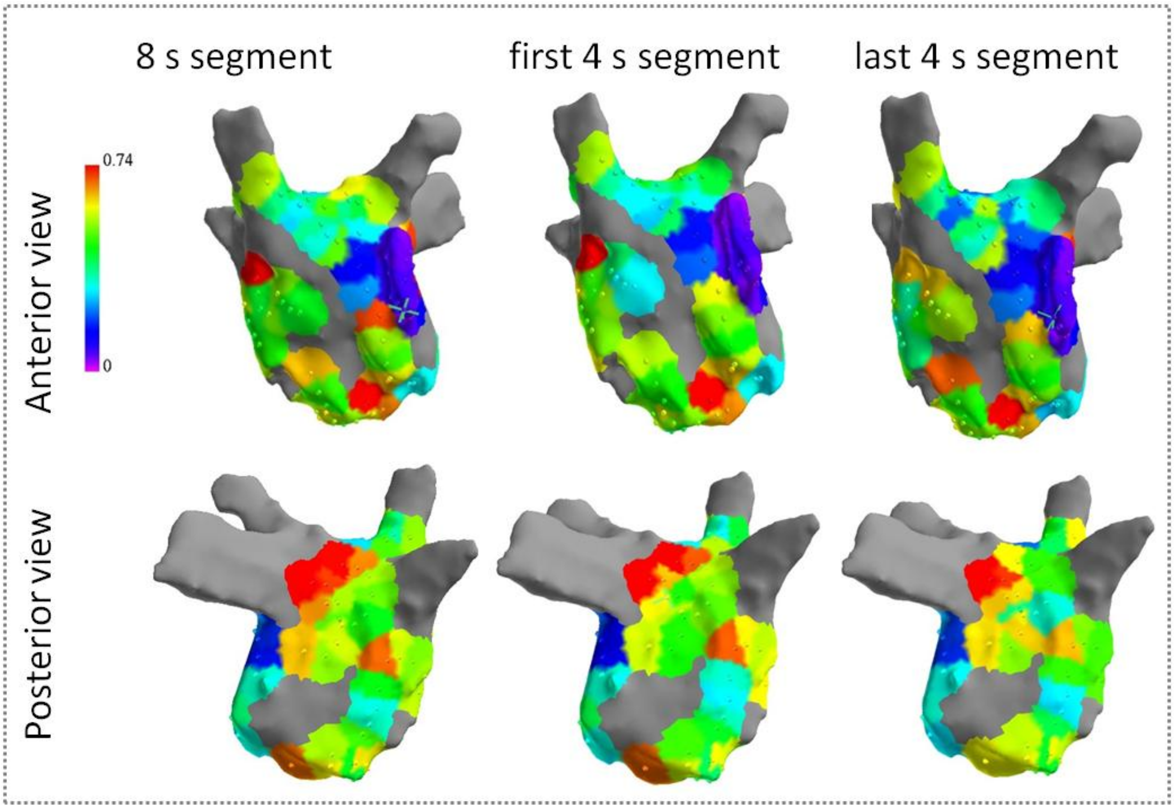

8 s segment
first 4 s segment
last 4 s segment
Anterior view
Posterior view
0.74
0

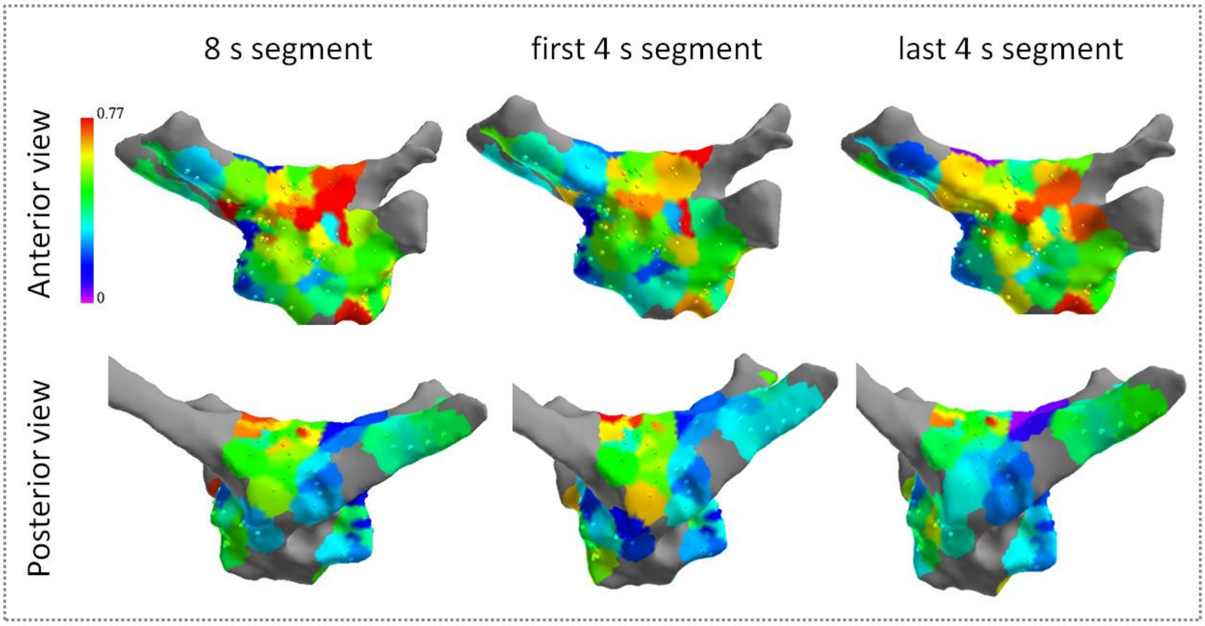

8 s segment
first 4 s segment
last 4 s segment
Anterior view
Posterior view
0.77
0

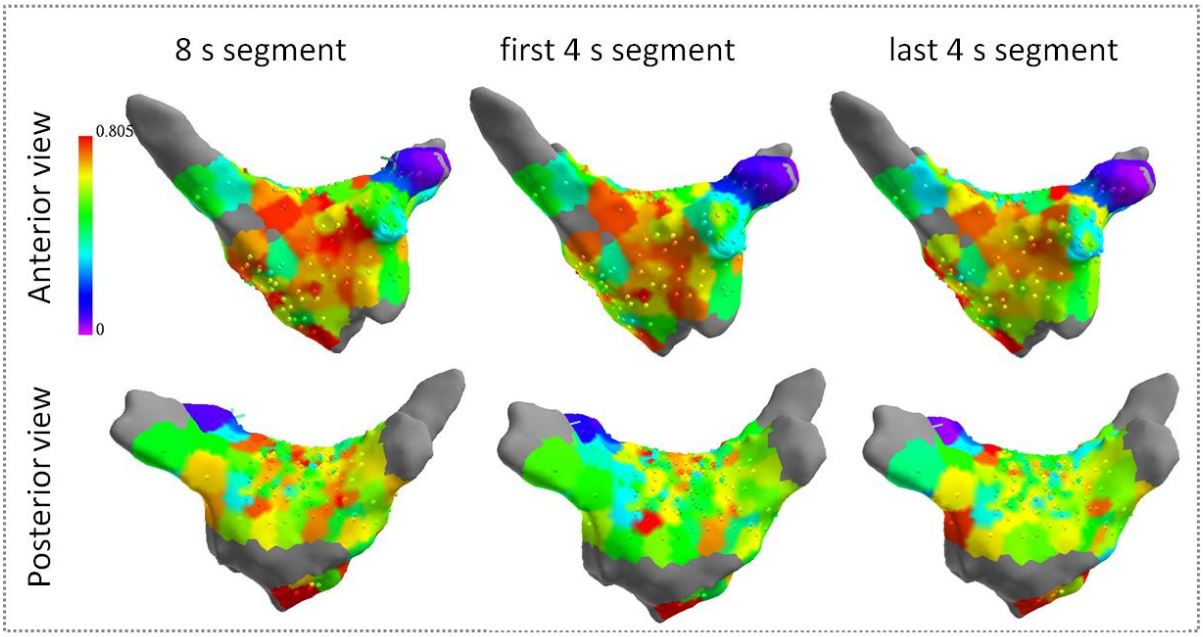
8 s segment
first 4 s segment
last 4 s segment
Anterior view
Posterior view
0.805
0

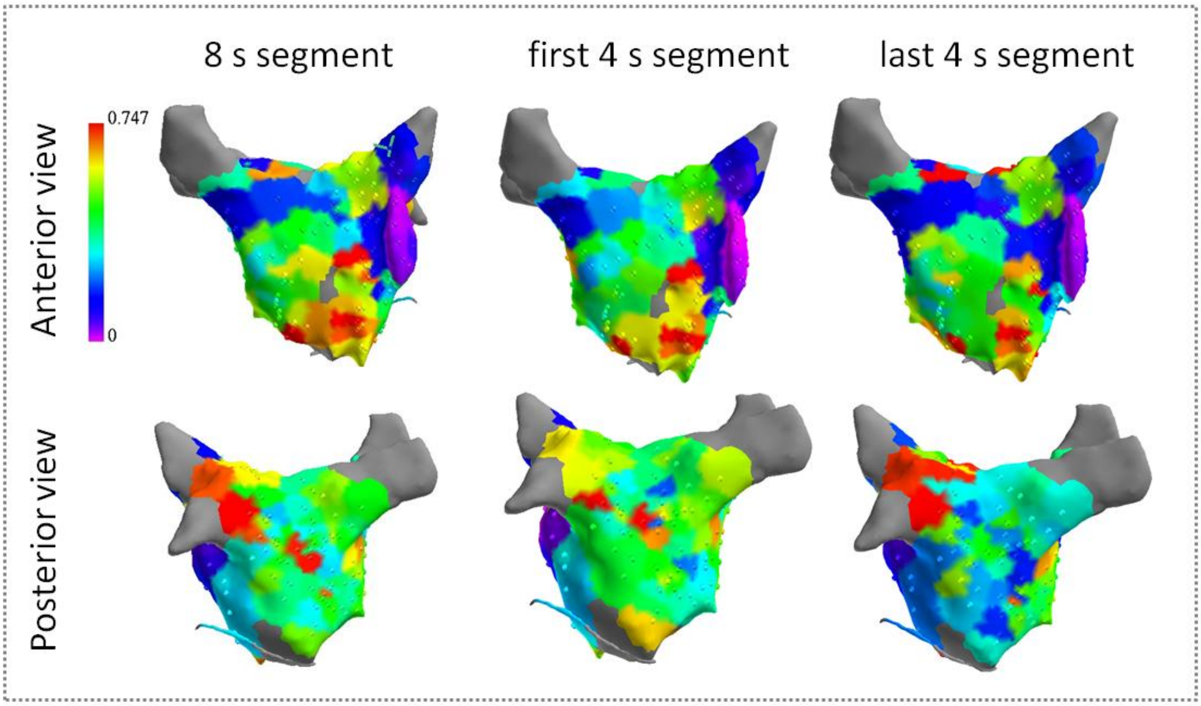
8 s segment
first 4 s segment
last 4 s segment
Anterior view
Posterior view
0.747
0

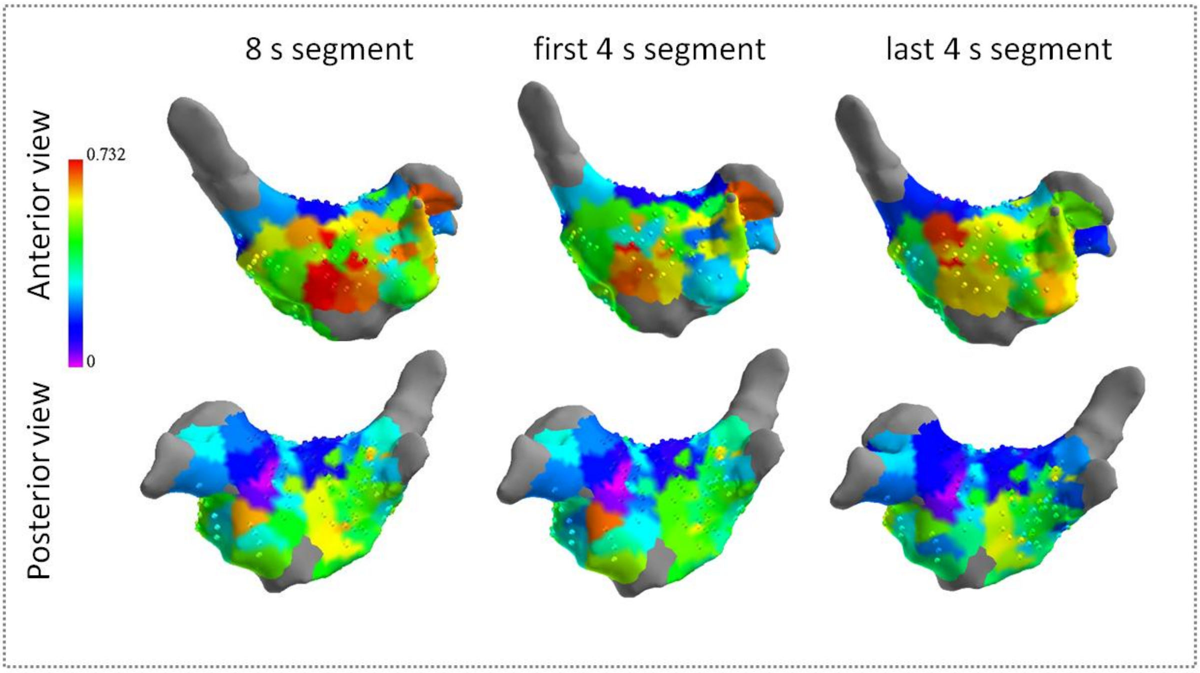

S3 Fig. Examples of temporal stability of dyssynchrony map (5 patients). Dyssynchrony map (anterior and posterior views) of whole 8 s electrograms segment on the left side. Map of the first 4 s segment in the middle and the map of last 4 s on the right side of the panel.
