# Supplementary material for: Local Electrical Dyssynchrony during Atrial Fibrillation: Theoretical Considerations and Initial Catheter Ablation Results
Source: PLoS One. 2016 Oct 25;11(10):e0164236. doi: 10.1371/journal.pone.0164236 (PMC5079563; doi:10.1371/journal.pone.0164236)
Supplement: S1 Text — (PDF) [file pone.0164236.s006.pdf]

Details on computation of electrogram phase

The first step in assessing the coherence of electrical activity between electrograms is a calculation of the electrogram phase. Phase, in the case of an oscillating system, is defined as a value denoting at which point of the cycle system is at given time point (see Figure A1). In the case of contact electrograms, a cycle is defined by consecutive local deflections (Figure A2.a). Phase reconstruction of atrial contact electrograms is challenging due to complex morphology which results in consequently complex morphology of the reconstructed phase (see Figure A2.b). This results in an extreme sensitivity to electrogram fractionation. To overcome this obstacle, we previously introduced a concept of sinusoidal recomposition which coupled with Hilbert transform was shown to be able to robustly reconstruct electrogram phase (1). In short, sinusoidal recomposition constructs a new signal which is a sum of sinusoidal wavelets with a period set to base cycle length of AF and amplitude proportional to the negative slope of the electrogram. Then, electrogram phase was defined as:

$$\varphi(t) = \arctan\left(\frac{-(u(t) - u^*)}{H(u)(t) - u^*}\right)$$

where $u(t)$ is recomposed signal, u^* denotes the mean of u(t) and H(u) denotes the Hilbert transform of $u(t)$. The original formulation presented in (1) was developed to reconstruct phase of unipolar electrograms. In the case of an unipolar electrogram, a morphological feature denoting the timing of wave passing is the maximum negative slope of local deflection. In the case of bipolar electrograms, this morphological feature becomes the maximum amplitude of local deflection. Therefore, since in our study we used bipolar electrograms, we modified original algorithm so that amplitude of the sinusoidal wavelets generated during recomposition is not proportional to the slope but rather to the absolute value of the electrogram.

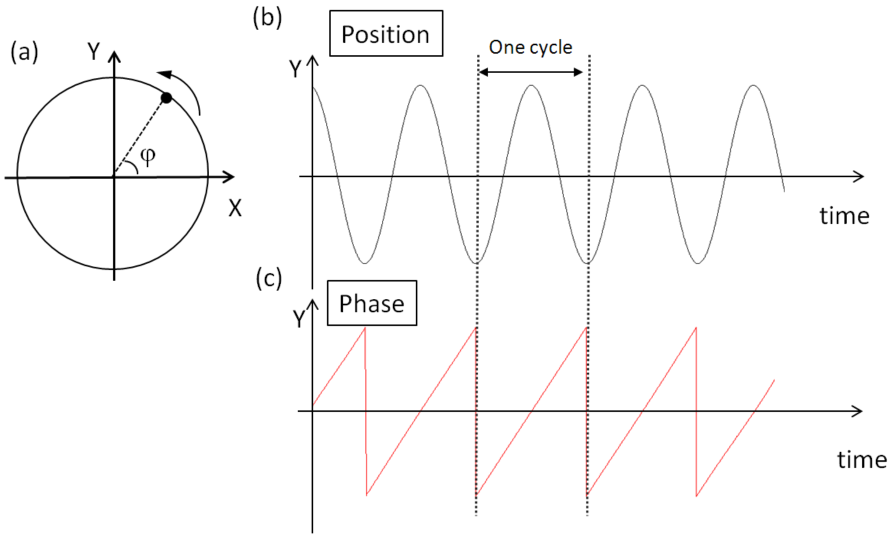

Figure A1 Definition of the instantaneous phase using the motion of an object along the circle as an example (a). The position of the object along the Y axis (b). Corresponding instantaneous phase (c). Completion of one full rotation corresponds with a change of the phase from a minimum to maximum value.

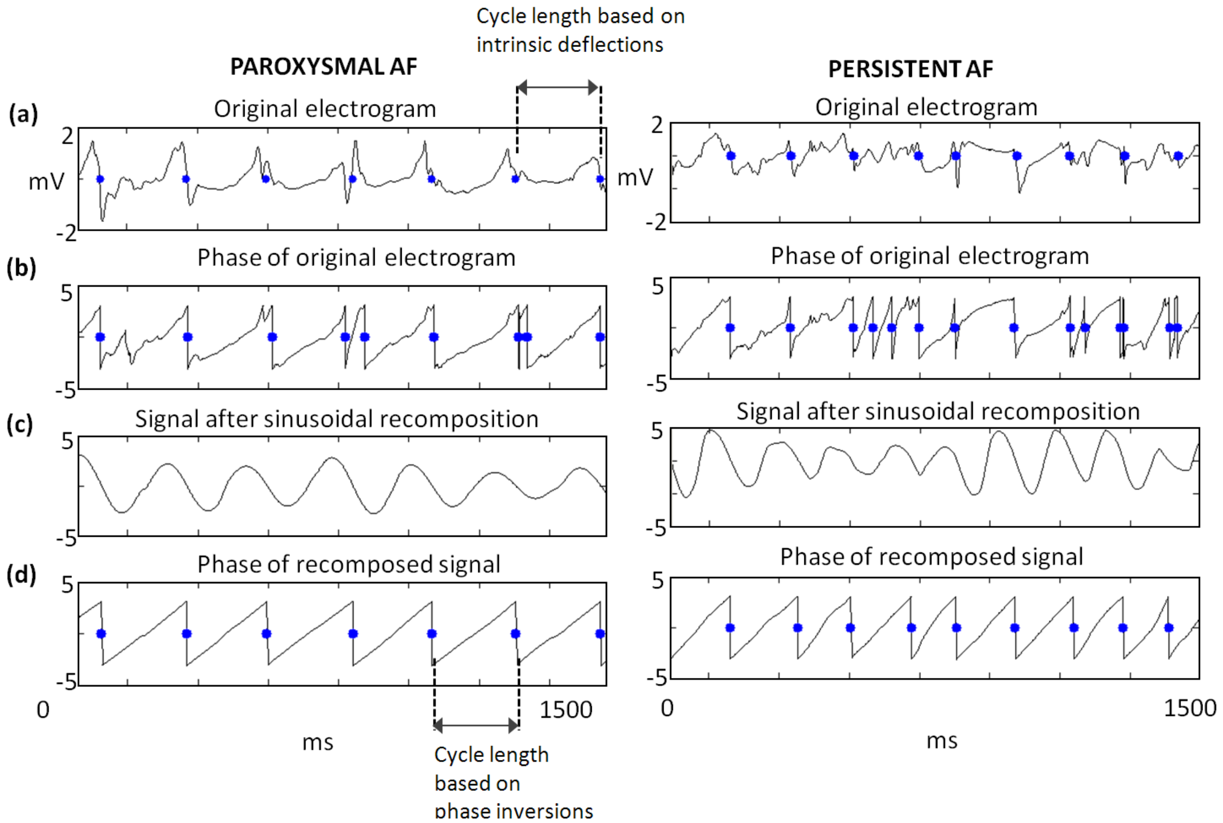

Figure A2 Phase reconstruction of contact atrial electrogram. Example electrograms from paroxysmal AF (left) and persistent AF (right) (a). Corresponding phase obtained using Hilbert transform in (b). Signal after application of sinusoidal recomposition to electrograms (c). The phase of recomposed signal (d).

References

1. Kuklik P, Zeemering S, Maesen B et al. Reconstruction of instantaneous phase of unipolar atrial contact electrogram using a concept of sinusoidal recomposition and Hilbert transform. IEEE transactions on bio-medical engineering 2014.
