# Supplementary material for: Local Electrical Dyssynchrony during Atrial Fibrillation: Theoretical Considerations and Initial Catheter Ablation Results
Source: PLoS One. 2016 Oct 25;11(10):e0164236. doi: 10.1371/journal.pone.0164236 (PMC5079563; doi:10.1371/journal.pone.0164236)
Supplement: S2 Text — (PDF) [file pone.0164236.s007.pdf]

Catheter ablation study details

Pts presenting to our institution for catheter ablation of persistent AF were eligible for this study if they fulfill the following inclusion criteria: (1) AF episodes lasting > 48 hours and < 24 months, (2) Episodes requiring external electrical cardioversion for restoration of sinus rhythm, (3) Age < 80 years, (4) Failure of at least 1 antiarrhythmic drug before ablation to maintain sinus rhythm and. Pregnant women were not included.

Conversion from AF to atrial tachycardia was defined as the occurrence of an organized atrial rhythm with a consistent atrial activation sequence in electrograms recorded at left atrial appendage, coronary sinus, and RA and by a monomorphic P-wave morphology.

**Age [y]**	59±9
**Male**	12/18
**AF history [y]**	3.1±2.2 (+2 pts with history > 10 y)
**Longest AF episode [m]**	4.4±7.5
**LA enlargement***	severe: 4/18, mild: 9/18
**Unsuccessful cardioversion**	8/18
**LV EF**	below 30%: 1/18, 40-55%: 1/18
**Hypertension**	8/18
**Coronary artery disease**	2/18
**Hyperlipidemia**	6/18
**Diabetes**	3/18
**Hypertrophic obstructive cardiomyopathy**	1/18
**AAD: prior ablation**	Amiodarone: N=5, β-blocker: N=15, Dronedarone: N=4, Propafenon: N=1
**AAD: post ablation**	Amiodarone: N=11, Flecanide: N=3, β-blocker: N=17, Dronedarone: N=1, Propafenon: N=3

Table B1 Patient characteristics (catheter ablation cohort). * LA enlargement classified following the definition in (1). AAD: anti-arrhythmic drugs.

**AF induction**	7/18
**Termination during PVI**	2/18
**Termination during dyssynchrony ablation**	7/18
**Mean AFCL at beginning of the procedure [ms]**	LAA :178±29, CS: 190±27, HRA: 202±55
**Mean AFCL after PVI [ms]**	LAA: 188±39, CS:194±32, HRA:193±47
**Mean AFCL after dyssynchrony regions ablation [ms]**	LAA:203±35, CS:207±32, HRA: 200±35
**AFCL increase during PVI [ms]**	LAA:13±20, CS:8±18, HRA: 1±27
**AFCL increase during dyssynchrony ablation [ms]**	LAA: 16±13, CS:18±9, HRA:17±37
**Procedure duration**	237±66 min
**Mean ablation time**	6272±2527 s

Table B2 AF ablation procedural characteristics and results. LAA: left atrial appendage, CS: coronary sinus, HRA: high right atrium.

References:

1. Lang RM, Bierig M, Devereux RB, Flachskampf FA, Foster E, Pellikka PA, et al. Recommendations for chamber quantification. European journal of echocardiography : the journal of the Working Group on Echocardiography of the European Society of Cardiology. 2006;7(2):79-108.
